# Disease modification in Parkinsonism: obstacles and ways forward

**DOI:** 10.1007/s00702-022-02520-6

**Published:** 2022-06-13

**Authors:** M. Höllerhage, M. Klietz, G. U. Höglinger

**Affiliations:** Department of Neurology, Hannover Medical School, Carl-Neuberg-Str. 1, 30625 Hannover, Germany

**Keywords:** Parkinson’s disease, Multiple System Atrophy, Progressive Supranuclear Palsy, Prodromal Parkinsonism, Biomarkers, Disease modification

## Abstract

To date, the diagnoses of Parkinson syndromes are based on clinical examination. Therefore, these specific diagnoses are made, when the neuropathological process is already advanced. However, disease modification or neuroprotection, is considered to be most effective before marked neurodegeneration has occurred. In recent years, early clinical or prodromal stages of Parkinson syndromes came into focus. Moreover, subtypes of distinct diseases will allow predictions of the individual course of the diseases more precisely. Thereby, patients will be enrolled into clinical trials with more specific disease entities and endpoints. Furthermore, novel fluid and imaging biomarkers that allow biochemical diagnoses are under development. These will lead to earlier diagnoses and earlier therapy in the future as consequence. Furthermore, therapeutic approaches will take the underlying neuropathological process of neurodegenerative Parkinson syndromes more specific into account. Specifically, future therapies will target the aggregation of aggregation-prone proteins such as alpha-synuclein and tau, the degradation of pathological aggregates, and the spreading of pathological protein aggregates throughout the brain. Many of these approaches are already in (pre)clinical development. In addition, anti-inflammatory approaches are in development. Furthermore, drug-repurposing is a feasible approach to shorten the developmental process of new drugs.

## Introduction

Since James Parkinson described Parkinson’s disease (PD) more than two centuries went by (Parkinson 1817; 2002). Not much changed in the diagnostic process since then. PD is considered a mainly clinical diagnosis (Postuma et al. 2015). Nevertheless, to date, we know much more about Parkinsonism then James Parkinson did in 1817. We know, for example, that Parkinsonism is not only caused by PD but also by other neurodegenerative disorders, considered as atypical Parkinson syndromes (aPS) (Levin et al. 2016a). Furthermore, since the discovery of alpha-synuclein (αSyn) as the main component of Lewy bodies in the late 1990’s (Spillantini et al. 1997), huge progress has been made in the understanding of the pathophysiological processes behind PD and related disorders. Now, we are able to distinguish between synucleinopathies and tauopathies histopathologically, dependent on the type of proteinaceous inclusions found in post-mortem tissue (Dickson 2018; Koga and Dickson 2018; Kovacs et al. 2020). Furthermore, genetic studies revealed that mutations in distinct genes are causative for a small two-digit percentage of cases of diseases previously considered as being sporadic (Bardien et al. 2011; Beavan and Schapira 2013). All these insights in the pathophysiology of Parkinson syndromes (PS) cannot hide that levodopa is still the most effective and best-tolerated drug to treat motor symptoms symptomatically in 2022. Furthermore, there is no drug with a proven disease-modifying effect for any of the distinct Parkinson syndromes. Moreover, non-motor symptoms present in PD and related disorders are more difficult to treat and levodopa is far less effective in many cases of aPS compared to PD.

In the following narrative review, we will discuss the obstacles on the way to disease modification in Parkinsonism. This includes the way how we make the diagnosis and the present absence of objective fluid and imaging biomarkers to confirm the diagnosis and to prove disease modification. Furthermore, we will present ongoing developments for clinical diagnostic criteria for early or even premotor stages of Parkinsonism, novel biomarkers, and ongoing and future therapeutic approaches that will shift the focus away from mainly symptomatic therapy towards the modification of the underlying pathophysiological processes of different diseases with Parkinsonism. Finally, we will discuss some obstacles in clinical care for patients with Parkinsonism.

## Diagnostic and pathophysiological aspects of Parkinson’s disease and atypical Parkinson syndromes

### Clinical differential diagnosis of Parkinson’s disease and atypical Parkinson syndromes

In clinical routine, the diagnoses of neurodegenerative PS are based on distinct diagnostic criteria. These usually consist of core criteria, supportive criteria, and exclusion criteria (Postuma et al. 2015; Höglinger et al. 2017; Wenning et al. 2022). However, these clinical criteria only allow the prediction of the underlying pathology with a certain probability. To make the definite diagnoses, a post-mortem neuropathological examination of the brain is necessary, which is still the gold standard for the diagnoses of neurodegenerative PS (Kovacs et al. 2020; Dickson 2018; Koga and Dickson 2018).

Histopathologically, PS can be divided in synucleinopathies and tauopathies, according to the presence of aggregates of alpha-synuclein (αSyn) or tau (Levin et al. 2016a). While PD, dementia with Lewy bodies (DLB), and multiple system atrophy (MSA) are all associated with the intracellular inclusions of misfolded αSyn, there are huge differences in the nature and localization of αSyn aggregates present in these disorders (Ayers et al. 2022; Koga et al. 2021). PD and DLB are characterized by the presence of Lewy bodies, which are large cytoplasmic deposits of αSyn in neurons (Goedert et al. 2013). In PD, in early stages, these are located in the brain stem, before they spread throughout the brain in the course of the disease (Braak et al. 2003), while the localization of (cortical) Lewy bodies in DLB is more diffuse (Koga et al. 2021). Moreover, the structure of brainstem and cortical Lewy bodies is different (Koga et al. 2021). MSA on the other hand is characterized by the presence of αSyn deposits in oligodendrocytes, called glial cytoplasmic inclusions (Koga et al. 2021). Progressive supranuclear palsy (PSP) and corticobasal degeneration (CBD), are characterized by the accumulation of 4-repeat tau in neurons and astrocytes (Rösler et al. 2019; Kovacs et al. 2020; Zhang et al. 2020). Targeting the different neurodegenerative processes will be essential for future neuroprotective therapies to slow or even halt the neurodegenerative process (Respondek et al. 2019). However, especially in early disease stages, the diagnostic accuracy to make the diagnosis of PD is low even if the diagnosis is made by movement disorder specialists (Adler et al. 2014). Moreover, the diagnostic accuracy further decreases, when the patients show an insufficient response to initial dopaminergic treatment (Adler et al. 2014). Furthermore, the sensitivity of the previous MSA diagnostic criteria was low at first visit (Osaki et al. 2009). Moreover, recent diagnostic criteria for PSP and MSA allow to make diagnoses at earlier staged by the introduction of “suggestive of PSP” (Höglinger et al. 2017) or “possible prodromal multiple system atrophy” (Wenning et al. 2022) as diagnostic certainty levels. However, there is always a trade-off between sensitivity and specificity. Therefore, the prediction of the specific neurodegenerative process by clinical examination alone can be very challenging, emphasizing the need for objective biomarkers to support the diagnostic process.

Furthermore, even typical PD motor symptoms including bradykinesia and rigidity are often misjudged as age-related or orthopedic in the beginning of the disease and it sometimes takes years until the correct diagnosis is made (Gaenslen et al. 2011). Thus, patients that are included in clinical trials that investigate the neuroprotective or disease-modifying potential of new agents might be too far progressed in the course of the disease to allow the observation of relevant changes in the progression rate.

In all neurodegenerative disorders, the pathophysiological process starts years or even decades before the occurrence of first symptoms. In PD, first motor-symptoms occur, when already 50% of the dopaminergic cells in the substantia nigra pars compacta died and 70–80% of striatal dopamine levels are depleted (Fearnley and Lees 1991). Furthermore, neuropathological examinations show that the substantia nigra is not the first structure to be affected by degeneration in PD, emphasizing that the pathophysiological process starts long before first motor symptoms occur (Braak et al. 2003).

### Specific subtypes in Parkinson’s disease and atypical Parkinson syndromes

In general, practice patients with Parkinson’s disease were grouped in tremor-dominant (TD), akinetic-rigid (AR) and equivalence type (ET) (Rajput et al. 2009). In PD patients with ET, tremor and akinetic symptoms are present to a similar extent. However, the classification in different subtypes of PD is very imprecise and can change over the course of the disease. Therefore, in the last decade, several groups spent effort in a more precise subtyping system of PD phenotypes (for critical review (Mestre et al. 2021). The more precise definition of subtypes of PD would help with the prediction of clinical disease progression, which would be beneficial for patient counselling. Furthermore, a more precise knowledge of the longitudinal course of the disease would be helpful in the recruitment for clinical trials and would allow recruiting of smaller groups of patients with a more homogenous progression. For the stratification in subtypes motor symptoms (e.g. more severe symptoms, less pronounced levodopa response), non-motor symptoms (e.g. orthostatic hypotension, cognitive decline, REM-sleep behavior disorder (RBD), and others), demographic markers (age at onset, disease duration), and other biomarkers may be used.

Hence, in a longitudinal study of pathological proven PD cases, the initial presentation of TD subtype, ET subtype, and the AR subtype determined disease progression (Rajput et al. 2017). In the study of Rajput et al., the TD subtype was associated with a more benign course of the disease, while the AR subtype showed the most malignant disease trajectory with shortest survival and highest rate of dementia (Rajput et al. 2009). Furthermore, the AR subtype was associated with more pronounced striatal dopamine depletion measured by post-mortem high-performance liquid chromatography compared to TD and ET subtypes (Rajput et al. 2008).

In another cohort of autopsy-confirmed PD patients, Pablo-Fernandez et al. defined subtypes of PD according to specific motor- and non-motor symptoms (Pablo-Fernández et al. 2019). By this approach, three different subtypes of PD with different rates of progression to specific milestones could be defined: the mild-motor, the intermediate, and the diffuse malignant phenotype. All these phenotypes showed a distinct pattern of Lewy body pathology in the brain (Pablo-Fernández et al. 2019). Interestingly, Alzheimer’s disease (AD) co-pathology was not significantly different between the subgroups of PD patients, although the authors observed an age-dependent accumulation of AD pathology in the cohort.

In an early study in this field, two subtypes of PD were determined mainly by motor symptoms in de novo PD patients and early stage PD patients on treatment: the tremor-dominant (TD) subtype and the postural instability and gait difficulties (PIGD) subtype (Jankovic et al. 1990). Already in 2010, a review of several studies identified the existence of several PD subtypes (PIGD, TD, old-onset and rapid progression, and young-onset slow progression) using cluster analysis (van Rooden et al. 2010).

In a recent study of PD subtypes, a biomarker-based approach revealed that plasma neurofilament light chain (NFL) was increased in the diffuse malignant subtype and was also associated with more rapid clinical progression of symptoms (Pilotto et al. 2021). In several cohorts, low amyloid beta 1-42 in the cerebrospinal fluid (CSF) was associated with faster cognitive decline in PD (Stewart et al. 2014). Interestingly, the PIGD subtype of PD was associated with lower amyloid beta 1-42 and higher P_181_-tau in the CSF compared to the TD subtype (Kang et al. 2013). And also in a more recent study, the diffuse malignant PD subtype was associated with lower amyloid beta 1-42 and amyloid beta/total tau ratio in the CSF compared to other phenotypes (Fereshtehnejad et al. 2017). Moreover, the diffuse malignant phenotype was associated with a higher extent of atrophy in cerebral magnet resonance imaging (Fereshtehnejad et al. 2017). The authors of a recent German study reported that serum NLF levels were increased in the PIGD subgroup and that NFL levels significantly correlated with the Montreal cognitive assessment (MoCA) scale and the MDS-UPDRS part III in PIGD patients (Pötter-Nerger et al. 2022), emphasizing the diagnostic value of this biomarker.

Definition of specific subtypes may also have some implications on treatment regimens in clinical routine. In a recent review, strategies for differential therapy were described in detail (Marras et al. 2020).

Contrary to various subtypes in PD, there are only two major phenotypes in MSA, the cerebellar subtype (MSA-C), and the Parkinsonism subtype (MSA-P) (Fanciulli and Wenning 2015). However, in longitudinal cohorts, there was no evidence of different progression rates between the two subtypes (Foubert-Samier et al. 2020). MSA is a rapidly progressive neurodegenerative disease with relatively constant progression of symptoms in both subtypes (Pérez-Soriano et al. 2021). Clinically, the extent of autonomic symptoms and earlier onset of RBD appear to be predictors for more rapid disease progression (Low et al. 2015; Giannini et al. 2020). Recent data also highlight the value of NFL as biomarker for disease severity and disease progression (Palleis et al. 2020; Zhang et al. 2022b).

In the past, there was mainly one phenotype of PSP (Litvan et al. 1996). However, a retrospective analysis of pathologically confirmed cases demonstrated that only a quarter of patients showed the classical phenotype, also called Richardson-syndrome (Respondek et al. 2014). Therefore, in the new MDS diagnostic criteria for PSP, a classification in different PSP variants was established (Höglinger et al. 2017), taking into account different clinical presentations of pathologically confirmed PSP cases (Respondek et al. 2014; Lopez et al. 2016; Respondek and Höglinger 2016). These new phenotypes help to clinically identify more patients with 4-repeat tau pathology in vivo and allow inclusion of these patients in natural history studies and clinical trials. The new PSP phenotypes can be divided into cortical and subcortical phenotypes. PSP-speech-language, PSP-frontal, PSP-corticobasal syndrome are referred to the cortical phenotypes (Guasp et al. 2021). The subcortical spectrum contains the PSP-parkinsonism, PSP-progressive gait freezing, PSP-oculomotor subtype and PSP-postural instability (Guasp et al. 2021). One obstacle with the diagnostic criteria of PSP is that patients can qualify for multiple phenotypes according to their clinical symptoms. This issue was recently solved by the MAX (multiple allocation extinction)-rules, which help to allocate patients to only one distinct phenotype (Grimm et al. 2019). However, after applying the MAX-rules, in clinically diagnoses PSP patients, the PSP-Richardson syndrome phenotype is relatively predominant compared to other phenotypes (Frank et al. 2020). Nevertheless, the main idea of the MDS-diagnostic criteria is the identification of more patients with cerebral 4-repeat tauopathy and the influence of different phenotypes on the identification threshold appears to be low (Respondek et al. 2017). In an extensive neuropathological investigation, Kovacs et al. were able to show specific patterns of neurodegeneration and tau pathology in a variety of brain regions in PSP patients (Kovacs et al. 2020).

Patients with PSP-Richardson syndrome present a relatively constant worsening of the disease with an annual deterioration of about ten points in the PSP rating scale (Piot et al. 2020; Grötsch et al. 2021). However, there are only very few data available on disease progression in other phenotypes. For example, in PSP-progressive gait freezing, disease progression is slow and cognitive deficits were only mild in most of the clinical cases (Owens et al. 2016). In terms of longitudinal progression, the MoCA test but not the Mini mental state examination (MMSE) was able to detect cognitive impairments in very early PSP patients. However, these tests were both not able to discriminate between specific phenotypes (Pereira et al. 2022).

With regard to biomarkers, it remains unknown if NFL is able to discriminate between specific subtypes of PSP. Nevertheless, NFL can predict disease progression in PSP patients (Jabbari et al. 2017; Rojas et al. 2018). The most established use of biomarkers in this field is the differentiation between PSP-corticobasal syndrome (PSP-CBS) and AD-corticobasal syndrome (AD-CBS). Amyloid beta 1-42 or ratios with total tau in CSF can be used to distinguish between PSP-CBS and AD-CBS (Höglinger et al. 2017). In recent years, also tau-PET imaging showed robust discrimination between PSP-CBS and AD-CBS (Palleis et al. 2021).

### Early to prodromal diagnosis of Parkinson’s disease and atypical Parkinson syndromes

As stated above, the clinical diagnosis of PD or aPS is generally based on clinical features. These features develop in the course of the disease. However, the presence of clinical motor-symptoms indicates an already advanced stage of the neuropathology. In PD for example, already 40–70% of substantia nigra dopaminergic neurons have died at time of diagnosis (Fearnley and Lees 1991). Therefore, in recent years, movement disorders experts put their focus more into prodromal or even preclinical phases of PD (Berg et al. 2015; Postuma and Berg 2016). Non-motor symptoms that precede the typical motor features of PD became more important, including hyposmia, depression, REM-sleep behavior disorder, and others (Postuma and Berg 2016). The first notion of RBD as a predictor of a future synucleinopathy was made in a cohort of RBD patients, in which the vast majority of patients developed PD or MSA over the time of a decade (Boeve et al. 2001; Iranzo et al. 2021).

Further work established constellations of specific symptoms e.g. mainly non-motor-symptoms of PD, which pre-manifest to PD motor symptoms (Heinzel et al. 2019). These symptoms were classified with their predictive value in the MDS PD risk calculator, which is available as an online tool on the MDS website (Heinzel et al. 2019). The likelihood of developing PD is mainly driven by a polysomnographic diagnosed RBD. However, there were several other factors driving the individual risk for the development of PD. A recent international multicenter study assessed the predictive value of additional factors for the pheno-conversion of RBD to a neurodegenerative synucleinopathy (PD, DLB, MSA). The factors with the greatest hazard ratio were quantitative motor testing or objective motor examination using the Unified Parkinson’s disease rating scale, followed by olfactory deficits, cognitive impairment, erectile dysfunction, motor symptoms, and an abnormal dopamine transporter SPECT (Postuma et al. 2019). Also others found that slight motor signs and abnormal dopamine transporter SPECT have a high predictive value for the development of PD (Simonet et al. 2021; Chahine et al. 2021a). Orthostatic hypotension had an intermediary predictive value, urge symptoms, constipation, and hyposmia had a very low predictive value (Shi et al. 2021). Using this risk assessment, premotor cohorts of PD can be built and early disease biomarkers may be further established (Iranzo et al. 2021). Still, one of the most relevant questions that remains unanswered so far is the prediction of the time point of pheno-conversion to motor PD (Miglis et al. 2021; Zhang et al. 2022a). Arnaldi and colleagues recently developed an additional tool for patient stratification to predict pheno-conversion to motor stages of PD (Arnaldi et al. 2022).

In stark contrast to the widely established and validated concept of recognizing PD in premotor stages, this concept is far less established in aPS. In the MDS diagnostic criteria for PSP, the new level of certainty “suggestive of” may help to make an earlier diagnosis of PSP (Höglinger et al. 2017). In a recent retrospective study using autopsy-confirmed cases of PSP, the time to the correct diagnosis could be reduced by the application of these criteria (Grimm et al. 2020). However, these data have to be confirmed in a prospective manner, which will be done in national PSP cohorts in Germany (Respondek and Höglinger 2021). Unfortunately, also, the criteria suggestive of the diagnosis of PSP rest up on motor features. A recent study tried to establish earlier symptoms of PSP patients by looking at general practitioner’s data (Viscidi et al. 2021). These data indicated that patients that later develop a PSP have more consultations because of movement disorders, gait disorders, falls, cognitive symptoms and depression compared to a matched control group. Furthermore, these patients more often complained about speech and visual problems (Viscidi et al. 2021). This study provides useful data for further studies on earlier identification of PSP patients.

In the new diagnostic criteria for multiple system atrophy, a novel “possible prodromal MSA” stage was established (Wenning et al. 2022). This stage mainly relies on the presence of RBD and has to be prospective evaluated for sensitivity and specificity of detection of patients eventually developing possible or probable MSA. The assumption of including only RBD patients in this stage seems to be unspecific at first. However, in the future, biomarker enhanced methods may improve specificity.

In these early neuropathological disease stages of PD and aPS, neuroprotective or disease-modifying drugs would have the highest impact on reducing disease progression resulting in sustained quality of life of the patients.

The biggest challenge in the next decade will be the standardized detection of people at risk for the development of a neurodegenerative Parkinsonian syndrome. The incidence of patients with prodromal PD or aPS in the population is low and prodromal symptoms are mainly unspecific (Heinzel et al. 2019). Usually, prodromal PD or aPS patients do not consider consulting a neurologist at first. Therefore, biomarker driven approaches have to be developed. Biomarker approaches may include digital biomarkers, fluid biomarkers, imaging biomarkers, or a combination of these. In addition, training of other clinical disciplines will be important to raise attention to early premotor signs of Parkinsonian syndromes.

### Genetic aspects

In general, neurodegenerative synucleinopathies and tauopathies are considered to be sporadic diseases. Monogenetic forms caused by mutations in *SNCA*, encoding αSyn or *MAPT*, encoding tau are very rare (Magistrelli et al. 2021) and are, therefore, of low relevance for most of the patients. However, genome-wide association studies (GWAS) showed that distinct single-nucleotide-polymorphisms (SNP) increase the risk to develop neurodegenerative Parkinsonism. In PD, over 90 SNPs have been identified in the most recent meta-analysis of GWAS (Nalls et al. 2019). In a PSP GWAS, SNPs in four genes including *MAPT* were identified to be associated with a higher risk to develop PSP. One of the genes, in which distinct SNPs were associated with a higher risk to develop PSP was *EIF2AK3*, encoding for the protein PERK that is part of the unfolded protein response (Höglinger et al. 2011). In MSA so far, no GWAS unequivocally identified a risk SNP with a statistically significant p value. However, a recent MSA GWAS of only histopathologically confirmed MSA cases found SNPs that were borderline significant in close neighborhood to *ZIC1* and ZIC4 (Hopfner et al. 2022). Since GWAS in general identify common variants with a low risk, the presence of individual SNPs in a patient has no huge influence on the risk to develop PD or an aPS. However, the compilation of variants into a polygenic risk score can be used to determine the risk of distinct individuals (Dehestani et al. 2021). In the future, these data might be used to specifically enroll patients at risk into clinical trials. Furthermore, data about the accumulation of risk SNPs in the same biological pathway could help to implement therapies that are tailored to the specific biological situation of the individual patient. Furthermore, in PD, mutations in genes other than *SNCA* play an important role. 5–10% of PD patients have a heterozygous mutation in the gene *GBA*, encoding the lysosomal protein glucocerebrosidase (Beavan and Schapira 2013), and up to 6% of sporadic PD patients have a mutation in *LRRK2,* encoding leucine-rich repeat kinase 2 (Bardien et al. 2011). To date, these specific genetic constellations have not been taken into consideration in most clinical trials and they are not at all considered in routine clinical care.

### Pathophysiological aspects

The pathological aggregation of αSyn and tau are the main pathophysiological events that lead to cell death in neurodegenerative Parkinson syndromes. In synucleinopathies, small oligomers of αSyn that form on the way from monomeric αSyn to the large aggregates found in Lewy bodies, are considered to be toxic for the cells (Bengoa-Vergniory et al. 2017; Alam et al. 2019). Also, in tauopathies, oligomers of the tau protein are considered to be toxic (Niewiadomska et al. 2021). Protein degradation mechanisms that are involved in the degradation of pathological protein aggregates are the ubiquitin–proteasome-system (Türker et al. 2021), the autophagy-lysosome-system consistent of chaperone-mediated autophagy and macroautophagy (Fleming et al. 2022), and the unfolded protein response (Johnston and Samant 2021). For example, in *GBA* mutation associated PD, one pathophysiological concept is that the lack of glucocebrosidase function leads to the formation of complex sphingolipids, which impair lysosomal function and thereby reduce degradation of pathological αSyn aggregates (Taguchi et al. 2017). Furthermore, cell culture experiments showed that activation of macroautophagy rescued human dopaminergic neurons from αSyn induced cell death (Höllerhage et al. 2014, 2019). Moreover, it was shown that activation of PERK, a protein involved in unfolded protein response, the gene of which *(EIF2AK3)* was associated with PSP in a GWAS (Höglinger et al. 2011), rescued cultured human neurons *in vitr*o and spinal cord neurons in mice in vivo from tau-induced toxicity in tauopathy models (Bruch et al. 2017).

Furthermore, cell-to-cell transmission of distinct pathological species of αSyn (Jan et al. 2021; Brás and Outeiro 2021) and tau (Brunello et al. 2020) in a so-called “prion like manner” is considered to be the mechanism behind the progression of synucleinopathies and tauopathies throughout the brain (Uemura et al. 2020). Histopathological studies showed that PD pathology can be divided in six stages. While in stage one and two, pathology is limited to deeper brainstem structures and the olfactory bulb, in stage three and four the pathological process reaches the midbrain including the substantia nigra leading to the typical motor symptoms (Braak et al. 2003). A similar staging system was established for tau pathology in PSP (Kovacs et al. 2020).

### Biomarkers

In the past years, biomarkers for PD including real-time quaking induced conversion (RT-QuIC) from the CSF and other biosamples (Poggiolini et al. 2021), presence of (posttranslationally modified) αSyn in the CSF (Kwon et al. 2022), auto-antibodies against αSyn (Folke et al. 2021), αSyn content in exosomes (Jiang et al. 2020, 2021), and αSyn deposits in the skin (Doppler 2021), were extensively investigated. However, none of these markers is already established in clinical routine care and methods to evaluate pathological signals vary between different laboratories. Nevertheless, many of these biomarkers are very promising. The RT-QuIC was first used in the prion field (Candelise et al. 2017). Is this assay, pathological protein seeds from biomaterial of patients are used to induce aggregation of recombinant protein. By shaking, these aggregates are disrupted again and then act as newly formed seeds for further aggregation. The continuous aggregation can be visualized by fluorescence labelling of beta-sheets within the aggregates (Atarashi 2022). In Creutzfeld-Jakob disease, the RT-QuIC was included into the most recent diagnostic criteria (Hermann et al. 2021). In addition, in the field of synucleinopathies, the usage of the RT-QuIC assay is advancing in recent years. A recent study provides evidence for distinct conformations of αSyn in PD and MSA by RT-QuIC analysis (Shahnawaz et al. 2020), and also others showed that αSyn RT-QuIC is suitable to distinguish between PD and aPS (Bargar et al. 2021; Russo et al. 2021; Singer et al. 2021). Thus, in the near future, RT-QuIC will help to distinguish between PD and aPS earlier in the course of the diseases, which can be difficult by clinical assessment alone (see above). Furthermore, it has also been shown that αSyn RT-QuIC reveals a positive signal in the CSF of most patients with idiopathic RBD (iRBD), considered as prodromal stage of PD. In a small series 18 out of 18 (100%) patients with idiopathic iRBD had a positive αSyn RT-QuIC signal in the CSF (Rossi et al. 2020). In a larger series, 47 out of 52 (90%) patients with iRBD had a positive αSyn RT-QuIC signal in the CSF (Iranzo et al. 2021). These data suggest that eventually, RT-QuIC will help to make the diagnosis of PD in a prodromal stage, before the occurrence of typical motor symptoms. Another αSyn-based biomarker is the content of αSyn in brain-derived exsomes in the plasma. Recent studies showed that exosomal αSyn is elevated in PD but not in healthy controls, patients with MSA, or other neurodegenerative PS that are not associated with αSyn pathology. However, clusterin was elevated in patients with a primary tauopathy (Jiang et al. 2020, 2021). Interestingly, exosomal αSyn was also elevated in patients with iRBD (Jiang et al. 2020). These data suggest that the investigation of proteins in the exosomes will be useful to distinguish between synucleinopathies and tauopathies in the future. Furthermore, existing data suggest that exosomal αSyn is a suitable marker to detect prodromal stages of PD.

Moreover, it was shown that the detection of phosphorylated αSyn in the skin can support the diagnosis of PD (Doppler 2021) and that skin αSyn can be used to distinguish between PD and tauopathies (Giannoccaro et al. 2022). In addition, more recently, RT-QuIC was used to investigate pathological αSyn aggregates in the skin, suggesting that this method was able to discriminate between synucleinopathies and tauopathies (Martinez-Valbuena et al. 2022). Interestingly, pathological αSyn deposits in the skin were also found in 10 out of 18 patients with RBD and in 20 out of 25 patients with early PS, but none of the investigated healthy control persons (Doppler et al. 2017). These data suggest that skin biopsy can be useful to prove αSyn pathology in a part of the patients and potentially can help to select patients for disease-modifying therapies in the future.

Another unspecific biomarker for neurodegeneration that was established in the past years is neurofilament light chain (NLF) (Gaetani et al. 2019). This marker can be used to distinguish PD from aPS because of the larger amount of neurodegeneration in aPS compared to PD (Rojas et al. 2018; Zhang et al. 2022b). The most promising fluid biomarkers and their added value are displayed in Table 1.

**Table 1:** Biomarker diagnostic in Parkinsonism

Marker	Pathological process	Added value
NFL	Neuronal (axonal) damage	Discrimination of PD from aPS
Blood neuronal exosomes	Detection of alpha-synuclein and tau in blood neuronal exosomes	Detection of synucleinopathies and tauopathies
RT-QuiC	Oligomerization of alpha-synuclein	Detection of synucleinopathies
A-beta 42, t-tau, p-tau and ratios	Amyloid and tau pathology	Exclusion of AD pathology in CBS, prediction of cognitive decline in DLB

### Imaging

Today, neuroimaging is one of the most essential diagnostic instruments to distinguish different etiologies of Parkinsonism. In a recent instructive review of the literature, Peralta et al. discuss imaging modalities and their contribution to making the differential diagnoses of PD and aPS (Peralta et al. 2022). The most relevant obstacle in neuroimaging is the detection of the underlying pathology in patients with Parkinsonism. Of course, magnet resonance imaging can rule out symptomatic lesions and structural changes in the brain, but structural changes as consequence of the neurodegenerative process appear quite late in the progression of PD and aPS and also do not occur in all patients (Mahlknecht et al. 2010). Imaging modalities, therefore, have to focus more on functional aspects. One promising method may be MR spectroscopy allowing the detection of specific regional metabolic changes (Ciurleo et al. 2014). The major limitation of MR spectroscopy was the regional restriction to one or several regions of interest. With whole-brain MR spectroscopy, this limitation has been overcome and this technique may offer interesting insights in early stages of PD and aPS (Ding and Lanfermann 2015). In a pilot study, metabolic alterations in early PD patients were detected compared to healthy controls (Klietz et al. 2019a). In addition, quantitative cerebral microstructural MR imaging might offer promising insight in the pathophysiology of PD and aPS. In a recent study in early PD patients, a distinct profile of quantitative microstructural changes in PD patients compared to healthy controls was described (Klietz et al. 2021c).

However, the in vivo detection of a synucleinopathy or tauopathy by imaging is still challenging. In the past years, tau PET tracers became available for research purposes, allowing the in vivo detection of hyperphosphorylated and misfolded intracellular tau protein. While the first generation of tau tracers showed unspecific binding patterns (Villemagne et al. 2018), the second generation was promising in recent studies as cross-sectional imaging biomarker (Brendel et al. 2020). For patients with CBS the combination of tau and amyloid PET imaging proved effective in determination of the underlying 4-repeat tau or AD pathology (Palleis et al. 2021; Song et al. 2021). Nevertheless, these tracers are not available in the clinical routine diagnostic yet. Despite these advances in the imaging of tau pathology, imaging of αSyn in vivo is still not possible (Korat et al. 2021).

In the future, a combination of radiological and fluid biomarkers will allow making early and correct diagnoses of PD and specific aPS. This will improve patient counseling, selection of patients for specific therapies, and more efficient inclusion of suitable patients in clinical trials.

## Studies with the aim to investigate neuroprotection

Since a neuroprotective therapy would be most efficacious before the neurodegenerative process is too advanced, an inclusion of patients in clinical trials as early as possible in the disease process is desirable. However, due to the lack of established clinical features, biomarkers, or imaging parameters that predict the occurrence of a neurodegenerative PS with a high likelihood, all studies so far enrolled patient that already fulfilled the diagnostic criteria of the distinct disorders. Furthermore, subtypes of PD were not considered in most PD trials (Pagano et al. 2021; Rascol et al. 2011). Recent trials in PSP enrolled patients according to the previous NINDS-SPSP diagnosis criteria (Höglinger et al. 2021) or by the presence of postural instability and vertical gaze palsy (Dam et al. 2021) and thereby de facto only included patients with a Richardson-syndrome without taking into account that these only comprise a quarter of all PSP patients (Respondek and Höglinger 2016). In MSA, some trial only included patients with predominant Parkinsonism (Dodel et al. 2010), whereas other trials included patients with MSA-C and MSA-P (Bensimon et al. 2009; Levin et al. 2016b, 2019).

Since at time of diagnoses the pathophysiological process is already advanced, the observation of strong clinical effects by potentially disease-modifying drugs might be compromised. Given that in PD 50% of dopaminergic neurons and 70–80% of striatal dopamine are already demised before typical motor symptoms occur (Jankovic et al. 1990), only 50% of the originally present dopaminergic neurons are left to be rescued in clinically established PD. A hypothetical disease-modifying therapy that would be able to rescue 50% of the dopaminergic neurons that are left, would lead to a further decline to 25% of the originally present cells, if therapy started at the time of diagnosis of clinically established PD. This would be accompanied by further increase in disability. On the other hand, if a therapy started at a stage, where 90% of the cells would be still present, the rescue of 50% of dopaminergic neurons would lead to a decline to only 45% of the originally present dopaminergic neurons, meaning that motor symptoms would occur much later or only very mildly. However, the rescue of 50% of the neurons would be an extremely huge achievement and is probably beyond any realistic expectations in the near future. Nevertheless, with a rescue of only 25% of the neurons left, one probably cannot expect much of a clinical benefit in patient that already lost 50% of the cells at the beginning of the treatment, since a rescue of 25% of the cells left would mean that only 12.5% of the originally present cells would survive in the long run. PD and aPS have a disease course of many years before motor symptoms occur (Gaenslen et al. 2011; Postuma and Berg 2016). If one assumed a demise of 10% of the dopaminergic cells in PD every year, within 1 year, the part of dopaminergic cells would decline from 50 to 45% in clinically established PD, even though natural history data suggest that annual clinical decline is measurable. However, given that there are inter-individual differences, it is conceivable that even complete or almost complete rescue of all cells that would have died in 1 year by treatment with a neuroprotective agent, would not lead to a relevant clinical difference compared to untreated patients. Nevertheless, in most recent trials, the duration of the treatment was limited to 1 year (Pagano et al. 2021) (NCT01560754). Furthermore, it is possible that agents that interfere with the pathological process would not be effective immediately, but only after a delay. Assuming that compounds that reduce aggregation of αSyn or tau, would immediately prevent further aggregation of any protein, it is still possible that already existent toxic aggregates would still do further harm to the cells. In light of the abovementioned assumptions, a trial duration of one year in already clinically established patients is probably too short to be able to observe relevant clinical effects and longer treatment and observation phases would be desirable. In line with that, in a previous trial with MSA patients, there was a tendency to improvement in the treatment group towards the end of the treatment period (Levin et al. 2019), which theoretically could have been more pronounced, if the trial duration had been longer. Furthermore, in many trials, patients with symptomatic co-medication with rasagiline were enrolled. Therefore, mild symptomatic effects which could be stronger with less endogenous dopamine left might have diluted small neuroprotective effects On the other hand, it is difficult to enroll patients with clinically established Parkinsonism without therapy in a trial with a duration of longer than a year, because many of the participants would eventually need effective symptomatic treatment. Another obstacle might be the reliance on clinical outcomes. Symptomatic effects of the treatment may confound clinical readouts. Furthermore, clinical scores have at least some interrater variability (Richards et al. 1994). Therefore, novel, objective outcome measures would be helpful. In a previous PD trial, a digital, completely objective outcome measure was used, showing that digital outcome measures are feasible (Pagano et al. 2021). In future clinical trials, it will be important to include patients in prodromal stages of PS, when neurodegeneration is not too far advanced. In addition to clinical readouts, also novel digital, objective readouts should be considered. Furthermore, since neuroprotective therapies might take longer until they have a relevant effect in comparison to symptomatic therapies, longer treatment and observation periods are desirable.

### Novel neuroprotective approaches

In future approaches for neuroprotective therapies, the molecular mechanisms behind neurodegeneration will move more into focus. These will comprise the reduced formation of pathological aggregates of tau and αSyn, the increased degradation of pathological aggregates, and the inhibition of cell-to-cell spreading of pathological protein aggregates.

### Reduction of the formation of pathological protein aggregates

Since pathological aggregates of αSyn and tau are involved in the pathophysiology of neurodegenerative Parkinson’s syndromes, one approach for a disease-modifying therapy would be the reduction of the formation of these pathological aggregates. One approach to achieve this is the reduction of the translation of the underlying proteins αSyn and tau. The idea behind this concept is that the presence of too much aggregation-prone protein (i.e. αSyn or tau) increases the risk of the formation of toxic oligomers that are harmful for the cells (Alam et al. 2019; Niewiadomska et al. 2021). Therefore, recently, antisense-nucleotides (ASO) were investigated. These are small nucleic acid sequences with a homology to a target messenger RNA (mRNA). The formation of double-strand RNA, that is not physiological in eukaryotic cells, leads to the degradation of the targeted mRNA and thus reduced protein translation. Phase I clinical trials investigating ASOs against αSyn in MSA (NCT04165486), and tau in PSP (NCT04539041) are ongoing. Furthermore, also a clinical trial targeting LRRK2 with ASOs is ongoing (NCT03976349).

Since, tau aggregates in PSP are mainly consistent of 4-repeat tau, another therapeutical approach would be to modify the splicing process of *MAPT*, to shift the presence of tau-protein from 4-repeat- to 3-repeat isoforms. Recently, a reporter system was developed that allows screening for specific splicing modifiers of MAPT (Truong et al. 2021). Thus, in the future, pharmacological interventions that modify MAPT-splicing might be discovered and eventually used in clinical care.

In addition to approaches that target the translation of aggregation-prone proteins αSyn and tau, other approaches aim for the inhibition of the aggregation process itself. The green tea extract epigallocatechin gallate (EGCG) is one compound that inhibits the aggregation of αSyn and other aggregation-prone proteins (Fernandes et al. 2021). Recently, in an investigator initiated double-blind placebo-controlled trial, EGCG was investigated in patients with MSA over one year. Interestingly, EGCG treatment led to a significant reduction in atrophy in the striatum and the precentral gyrus in a subgroup of patients that were investigated with MRI scans. Furthermore, EGCG treatment led to a tendency in the reduction of clinical progression compared to placebo (Levin et al. 2019). Even though the clinical benefit was not statistically significant, data from the MRI subgroup and the tendency to clinical benefit are promising. For once, these data suggest that a treatment of one year might have been too short to observe a statistical significant clinical benefit. Furthermore, these data suggest that inhibition of aggregation is a feasible approach and therefore might be more effective, if used in earlier or prodromal disease stages in the future.

Another interesting anti-aggregatory compound is anle138b. The lead structure of this compound was identified in a cell-free screening assay, in which 20.000 compounds were investigated for their potential to reduce αSyn aggregation in vitro. After the primary screening, the lead structure was chemically modified in order to improve its anti-aggregatory properties (Wagner et al. 2013). Anle138b proved to be efficient in mouse models of PD (Levin et al. 2014), MSA (Lemos et al. 2020; Heras-Garvin et al. 2019; Fellner et al. 2016), tauopathies (Wagner et al. 2015), and Alzheimer’s disease (Brendel et al. 2019). Recently, a phase I-trial with healthy person investigating safety and tolerability of anle138b was successfully finished (NCT04208152) and further trials in patients are planned.

### Increased degradation of pathological protein aggregates

Another approach to reduce the burden of pathological protein aggregates is increased degradation. Mechanisms that are involved in the degradation of pathological αSyn and tau-aggregates are the ubiquitin–proteasome-system (Türker et al. 2021), autophagy, which comprises macroautophagy, chaperone-mediated autophagy, and microautophagy (Fleming et al. 2022), and the unfolded protein response (Johnston and Samant 2021). In macroautophagy, large protein aggregates and defective organelles are engulfed by a double-membrane structure called the autophagosome. In the process of macroautophagy the autophagosome is fused with the lysosome, in which degradation occurs (Feng et al. 2014). Activators of macroautophagy proved to be protective against αSyn induced toxicity in a PD cell model (Höllerhage et al. 2014, 2019). However, also reduction of the formation of autophagosomes as one of the first steps of macroautophagy protected dopaminergic neurons in vitro from αSyn-induced toxicity and bypassing of macroautophagy led to increased exosomal release of αSyn (Fussi et al. 2018). Thus, the role of macroautophagy as a therapeutic target is not yet fully understood. Previously, it was suggested that a pulsatile activation of macroautophagy might be a feasible approach as therapy for PD (Fowler and Moussa 2018). While autophagy is an essential intracellular process, an overstimulation might be harmful for the cells, which is supported by observations that mutated αSyn stimulated macroautophagy and thereby led to cell death (Choubey et al. 2011). However, others showed that wild-type αSyn led to impaired macroautophagy which was also harmful (Winslow and Rubinsztein 2011). In a pulsatile therapeutic regimen, by activation of autophagy one could intermittently remove pathological protein aggregates, while autophagy function would be left to its physiological states in the intervals.

In PD caused by heterozygous GBA mutations, one assumption is that impaired function of the lysosomal protein glucocerebrosidase leads to the formation of complex sphingolipids from glycosylceramide and these then lead to impaired lysosomal function. Consequently, toxic αSyn species accumulate and lead to cell death. Physiologically glycosylceramide is metabolized by glucocerebrosidase into ceramide and glucose. In a previous trial (MOVES-PD, NCT02906020), the glucosylceramide-synthase inhibitor venglustat was investigated in PD patients with a heterozygous GBA-mutation with the aim to lower the substrate for the formation of complex sphingolipids and thereby prevent impairment of lysosomal function and accumulation of toxic αSyn species. Interestingly, patients treated with venglustat showed a linear decline in motor function as measured by an increase of the score of part II and III of the MDS-UPDRS, while placebo-treated patients stayed stable first, before also a linear decline started. This result was interpreted as a mild negative symptomatic effect of venglustat. Another therapeutical approach in GBA-mutation associated PD would be the activation of glucocerebrosidase. Currently, a phase II trial with a glucocerebrosidase activator (BIA 28-6156 / LTI-291) is in preparation.

The unfolded protein response (UPR) is a cellular mechanism that is involved in protein quality control and degradation of misfolded proteins. Interestingly, *EIF2AK3*, the gene encoding for PERK, a protein involved in UPR, was identified as a risk gene for PSP in a previous GWAS (Höglinger et al. 2011). Furthermore, mutations in *EIF2AK3* lead Wolcott-Rallison-syndrome (WRS), an extremely rare condition with complete absence of PERK which is associated with neurodegeneration. Moreover, histopathological examination of a post-mortem WRS brain showed changes in autophagic flux in absence of PERK, suggesting an interplay between the UPR and autophagy (Bruch et al. 2015). Interestingly, experimental data showed that pharmacological activation of PERK rescued neuronal cells in in vitro and in vivo models of tauopathies (Bruch et al. 2017), suggesting that PERK activation might be a rational approach for a neuroprotective therapy of PSP and other tauopathies.

### Inhibition of spreading of aggregation-prone proteins

Cell-to-cell spreading in a so-called “prion-like” manner plays an important role in the progression of neurodegenerative disorders, including synucleinopathies and tauopathies, throughout the brain (Jucker and Walker 2018). Therefore, prevention of cell-to-cell spreading is a rational approach for disease-modifying therapies. Cell-to-cell spreading includes the release of toxic protein species from diseased cells, the presence of toxic protein species in the extracellular space, and the uptake of toxic protein species in previously healthy cells. All three parts are potential targets for neuroprotective therapies. In the past years, the understanding of the specific uptake mechanisms of pathological protein aggregates increased a lot. Previously it was shown that reduced levels of BIN1-amphysin2, a negative regulator of endocytosis led to increased tau propagation in cell culture (Calafate et al. 2016). Others found that heparin sulfate proteoglycans are involved in the uptake of tau-fibrils (Holmes et al. 2013). Recently, it was published that receptor-related protein 1 (LRP1), a member of the LDL receptors family is involved in tau uptake (Rauch et al. 2020). Furthermore, some studies suggest that receptors on the cell surface such as lymphocyte activating gene 3 (LAG3) and amyloid precursor-like protein 1 (APLD1) are involved in intracellular internalization of distinct αSyn species (Mao et al. 2016; Zhang et al. 2021). However, more recently, it was questioned, if LAG3 was expressed in human cells at all (Emmenegger et al. 2021). While approaches that target specific uptake mechanisms are still in early stages of preclinical development, approaches that aim on the scavenging of pathological protein species from the extracellular space using specific antibodies are more advanced. Previously, two phase II trials were conducted in PSP investigating the disease-modifying potential of tau-antibodies with an N-terminal epitope. However, both trials failed to meet the primary efficacy endpoint which was clinical improvement compared to placebo measured with the PSP rating scale (Höglinger et al. 2021; Dam et al. 2021). This led to the discussion, if either the epitope of the investigated antibodies was wrong, or the therapies came too late, or if tau was the correct target at all (Jabbari and Duff 2021). However, new tau antibodies with other tau epitopes are in the development pipeline, one of these (UCB0107, bepranemab), is already under investigation in early phase clinical trials (NCT04658199).

Furthermore, also in PD, trials with antibodies against αSyn were conducted. While, a trial with the N-terminal anti-αSyn antibody cinpanemab (NCT03318523) failed to meet primary and secondary endpoints, another trial with the C-terminal anti-αSyn antibody prasinezumab (NCT03100149) (Pagano et al. 2021) showed promising results with a trend towards improvement in the treatment group compared to the placebo group in the MDS-UPDRS part III. Furthermore, this trial is in particular interesting, because in addition to the MDS-UPDRS, a digital motor score was used as outcome parameter. Interestingly, in this score there was a significant improvement in the treatment group compared to placebo with a slightly higher effect in the group with the lower dose of prasinezumab. Nevertheless, these data are very interesting, because they show the feasibility of a completely objective bias-free readout in a clinical trial.

### Anti-inflammatory approaches

In the past years, the contribution of inflammation in neurodegenerative disorders came more into focus (Pajares et al. 2020; Nichols et al. 2019). Inflammatory processes in the central nervous system involve the activation of microglia. Amongst other causes, reactive oxygen species (ROS) generated by an enzyme called myeloperoxidase lead to activation of microglia. Therefore, previously a myeloperoxidase inhibitor (verdiperstat) was investigated in a clinical trial in MSA (NCT03952806). Even though the trial failed to meet its primary and key secondary outcome measures, anti-inflammatory approaches might play a role in future therapies of neurodegenerative PS.

### Drug repurposing

Another approach towards novel therapies for PS would be the repurposing of already approved drugs. Drug repurposing has the advantage of saving costly and time-consuming steps in the development of completely new drugs, since data about compatibility, dosage, and pharmacokinetics of approved drugs are readily available. For instance, we previously performed a drug-screening of 1,600 FDA-approved drugs in an αSyn cell model and identified that the phosphodiesterase I inhibitor, vinpocetine, protected human neurons from αSyn-induced toxicity in vitro and murine neurons in vivo (Höllerhage et al. 2017). Since vinpocetine is approved in many European countries for indications other than Parkinsonism or synucleinopathies, and the drug is generally well-tolerated, it would be reasonable to investigate a potential neuroprotective effect in patients suffering from a neurodegenerative synucleinopathy (e.g. iRBD, PD, or MSA).

## Clinical care for patients with Parkinson’s disease and atypical Parkinson syndromes

There are several obstacles in care for people with PD or aPS. In this part of the review, we will mainly focus on later disease stages, although we know that there are also several issues at the beginning of the disease as well. These are the psychological impact of communicating the diagnosis of prodromal PD or aPS to previously healthy individuals and management of their coping with this information.

### Patient management: drug safety

In advanced stages of PD and aPS, most of the patients are in a geriatric age and display several characteristics of geriatric patients like multimorbidity and polypharmacy (Greten et al. 2021; Klietz et al. 2019b). These patients are extremely vulnerable for side effects of drug treatment (Lingor et al. 2016). In PD, patients display a distinct profile of comorbidities including hypertension, heart failure, polyneuropathy, diabetes mellitus, sarcopenia, frailty, and cerebral small vessel disease (Greten et al. 2021; Klietz et al. 2019b). Treatment of these patients needs a careful monitoring of drug effects and side effects. In a recent review, a strategy for monitoring of these patients has been proposed (Klietz et al. 2019b). Furthermore, geriatric visits should include specific assessments and strategies including discontinuation of medication (Scott et al. 2015). The usage of drug prescribing lists to detect potentially inadequate medication can be helpful in these patient groups. In a recent study, the FORTA classification system has been described in geriatric PD in-patients as a promising tool to increase drug-safety (Greten et al. 2021). In two studies, typical comorbidities with high prevalence in PSP patients were arterial hypertension and diabetes (Papapetropoulos et al. 2005; Rabadia et al. 2019). This has also been reported in a recent study that used PSP patients’ data from general practitioners in Germany (Kwasny et al. 2021). Furthermore, German health care data report common cardiovascular, muscular, neurological, and urogenital comorbidities in PSP (Zella et al. 2020). However, typical drug constellations in the treatment of PSP and MSA patients are not clear and should be determined in larger cohorts. In summary, patients with advanced PD or aPS are more likely to present with geriatric strains like multimorbidity and polypharmacy. Furthermore, these patients often present with cognitive impairments (Klietz et al. 2019b). Therefore, these patients have a higher likelihood of medical emergencies and need drug-safety concepts (Moßhammer et al. 2016; Okunoye et al. 2020). In the future, drug safety concepts should be digital. However, in the meantime measures like the emergency box might help to cope with drug-safety issues in acute emergency referral (Krey et al. 2021).

### Palliative care

Patients with PD have an only mildly reduced life expectancy (Macleod et al. 2014). However, an increasing number of symptoms lead to reduced quality of life over a long disease duration. In patients with aPS life expectancy is dramatically reduced and also symptom burden severely reduces quality of life. The international multicenter prospective CLASP study examined late-stage PD and aPS patients and described an extensive reduction in the activities of daily living and quality of life (Balzer-Geldsetzer et al. 2018). Advanced PD and aPS patients suffer from severe motor and non-motor symptoms (Schrag et al. 2020). In particular, neuropsychiatric symptoms contribute to reduced quality of life (Hommel et al. 2020; Eichel et al. 2022). As also discussed earlier, there is still no cure for neurodegenerative PS. Therefore, palliative care approaches are extremely important in the holistic treatment of these patients (Miyasaki 2013; Miyasaki and Kluger 2015; Oliver et al. 2016). Unfortunately, only a very small proportion of patients with PD received palliative care interventions (Klietz et al. 2018; Safarpour et al. 2015). In a case series, Bükki et al. reported palliative care interventions in patients with aPS in a hospital setting. Most patients could be discharged after acute interventions with improved symptom control and increased quality of life (Bükki et al. 2016). Interestingly, about 72% of these advanced PD patients have an advance directive (Klietz et al. 2018). Investigation of the specific content of these advance directives revealed that the vast majority did not address disease specific complications (Klietz et al. 2019c). In a consensus-based Delphi panel of German palliative care and PD experts, disease specific recommendations for specific advance directives for people with PD have been developed (Klietz et al. 2020a). One explanation for the low-grade implementation of palliative care interventions in PD patients may be the low level of palliative care training in neurologists compared to general practitioners (Jensen et al. 2022). Cooperation between neurologists and palliative care specialists mainly focuses on patients with brain tumors and amyotrophic lateral sclerosis. Unfortunately, concepts for other progressive neurological diseases are rare (Oliver et al. 2020). In a recent international multicenter study, concepts for palliative care interventions were studied and palliative care training programs were developed (Gatsios et al. 2021; Meinders et al. 2021). A survey on aPS patients revealed that this patient group prefers an early conversation about diagnosis with information about the disease, natural history, prognosis, and treatment options (Saranza et al. 2021). These aPS patients also want to have a conversation about end of life and advance care planning in later stages of the disease (Saranza et al. 2021). Advance care planning concepts are necessary to improve clinical care (Lum et al. 2019; Seeber et al. 2019). Moreover, in aPS, early psychological interventions should be employed, because of the large proportion of patients undergoing assisted suicide (Nuebling et al. 2021; Lum et al. 2019; Churm et al. 2022). In the future, palliative care interventions must become an essential part of PD and, even more importantly, aPS therapy (Miyasaki et al. 2022). In addition, more neurologists need specific training in palliative care, not only for PD and aPS patients.

### Caregiver burden

Finally, to optimize patient care, the caregiver must also be considered. Caregiver burden is an extremely important issue in PD and also aPS (Mosley et al. 2017). In a recent study, caregiver burden and caregiver burnout was identified as a relevant factor contributing to transition to institutional care (Jensen et al. 2021). Furthermore, caregivers provide a huge quantity of informal care which is a huge economic factor in PD (Martinez-Martin et al. 2019). Measurement of caregiver burden can use generic scales such as the Zarit burden scale or disease specific scales such as the very well-validated Parkinson’s disease caregiver burden questionnaire (Zarit et al. 1986; Zhong et al. 2013; Klietz et al. 2019d, 2021a). The usage of disease specific questionnaires can assess the relationship of specific symptoms of a specific disease to the perceived caregiver burden. However, in the clinical routine, a fast assessment can also be performed by using visual analogue scales or other simple tools with good correlations to more detailed questionnaires (Klietz et al. 2019d; Jensen et al. 2021). A very interesting tool is the recently published caregiver task questionnaire, which allows to assess the perceived burden of specific caregiving tasks in a specific caregiver setting (Klietz et al. 2021b). Caregiver burden is mainly determined by the patient’s loss of autonomy and increasing care-dependency. General factors contributing to caregiver burden are exacerbated by the quantity of caregiving hours per day (Grün et al. 2016; Tessitore et al. 2018; Genç et al. 2019). More specific aspects influencing caregiver burden in PD were motor symptoms, cognitive decline, and other neuropsychiatric symptoms (Klietz et al. 2020b, c; Eichel et al. 2022; Bruno et al. 2016; Chahine et al. 2021b; Martinez-Martin et al. 2015). In the future, physicians must include caregiver burden and plans for interventions aiming to reduce this burden and allow sustained care at home in the evaluation of PD and aPS patients with complex symptoms. In patients with huge symptom load and caregiver burnout, a transition to institutional care might be a good option to reduce caregiver burden, improve overall quality of care for these patients, and even improve relationship satisfaction (Heine et al. 2021; Jensen et al. 2021; Trapp et al. 2019). Management programs for patients and their caregivers might improve coping with complex symptoms for both the patients and their caregivers (Hellqvist et al. 2018, 2020; Tennigkeit et al. 2020). Treatment of complex patients in specific PD networks may improve overall care and efficacy of PD treatment by including all relevant stakeholders (Prell et al. 2020a, b).

## Conclusion

Disease modification in Parkinsonism and other neurodegenerative conditions is still a holy grail, since there is no therapy yet with a proven disease-modifying effect. However, many very promising approaches are in development. Theses comprise the inhibition of the formation of pathological protein aggregates by lowering total protein levels or by pharmacological inhibition of the aggregation process. Furthermore, other approaches aim to reduce the burden of pathological protein aggregates by inducing their degradation. Other approaches target the cell-to-cell spreading of the neurodegenerative process by inhibiting the uptake of pathological protein aggregates on the one hand or by scavenging pathological protein species from the extracellular space using antibodies on the other hand. A different, less mechanistic approach is drug-repurposing with the advantage that time-consuming and costly steps in the development of new drugs can be skipped, because data regarding dosage, pharmacokinetics, and tolerability of already approved drugs are readily available. Furthermore, in the future, it will be of utmost importance to identify persons at risk to develop PD or a related disorder as early as possible to start the investigation of potentially neuroprotective treatments in a stage before neurodegeneration is too marked to achieve a relevant clinical effect. Tools like the MDS risk score as well as novel biomarkers can help with that. Furthermore, novel readouts including biomarkers representing the ongoing pathological process and bias-free digital clinical readouts will support the ongoing quest for disease modification in Parkinsonism. We are very optimistic that disease modification is on the verge to become reality and that we will experience huge changes in the way how we treat Parkinsonism within the 20’s of the twenty-first century. Nevertheless, despite all treatment options, there will still be large groups of patients reaching advanced disease stages in need for excellent clinical care. These patients will profit from drug-safety interventions, palliative care and interventions to reduce caregiver burden.
